# Immunization with short peptide particles reveals a functional CD8^+^ T-cell neoepitope in a murine renal carcinoma model

**DOI:** 10.1136/jitc-2021-003101

**Published:** 2021-12-03

**Authors:** Xuedan He, Shiqi Zhou, Melissa Dolan, Yuhao Shi, Jianxin Wang, Breandan Quinn, Dushyant Jahagirdar, Wei-Chiao Huang, Moriya Tsuji, Roberto Pili, Fumito Ito, Joaquin Ortega, Scott I Abrams, John M L Ebos, Jonathan F Lovell

**Affiliations:** 1 Department of Biomedical Engineering, University at Buffalo, Buffalo, New York, USA; 2 Department of Experimental Therapeutics, Roswell Park Comprehensive Cancer Center, Buffalo, New York, USA; 3 Center for Computational Research, University at Buffalo, Buffalo, NY, USA; 4 Department of Anatomy and Cell Biology, McGill University, Montreal, Québec, Canada; 5 Department of Medicine, Columbia University Irving Medical Center, New York, NY, USA; 6 Department of Medicine, State University of New York, Buffalo, NY, USA; 7 Department of Immunology, Roswell Park Comprehensive Cancer Center, Buffalo, New York, USA

**Keywords:** adaptive immunity, antigens, vaccination

## Abstract

**Background:**

Induction of CD8^+^ T cells that recognize immunogenic, mutated protein fragments in the context of major histocompatibility class I (MHC-I) is a pressing challenge for cancer vaccine development.

**Methods:**

Using the commonly used murine renal adenocarcinoma RENCA cancer model, MHC-I restricted neoepitopes are predicted following next-generation sequencing. Candidate neoepitopes are screened in mice using a potent cancer vaccine adjuvant system that converts short peptides into immunogenic nanoparticles. An identified functional neoepitope vaccine is then tested in various therapeutic experimental tumor settings.

**Results:**

Conversion of 20 short MHC-I restricted neoepitope candidates into immunogenic nanoparticles results in antitumor responses with multivalent vaccination. Only a single neoepitope candidate, Nesprin-2 L4492R (Nes2LR), induced functional responses but still did so when included within 20-plex or 60-plex particles. Immunization with the short Nes2LR neoepitope with the immunogenic particle-inducing vaccine adjuvant prevented tumor growth at doses multiple orders of magnitude less than with other vaccine adjuvants, which were ineffective. Nes2LR vaccination inhibited or eradicated disease in subcutaneous, experimental lung metastasis and orthotopic tumor models, synergizing with immune checkpoint blockade.

**Conclusion:**

These findings establish the feasibility of using short, MHC-I-restricted neoepitopes for straightforward immunization with multivalent or validated neoepitopes to induce cytotoxic CD8^+^ T cells. Furthermore, the Nes2LR neoepitope could be useful for preclinical studies involving renal cell carcinoma immunotherapy.

## Introduction

Renal cell carcinoma (RCC) is among the 10 most common cancers globally, with an incidence rising in the past decade.^1^ Likely due to its immunogenicity, RCC has been a prime target for cancer immunotherapies, initially with high-dose interleukin (IL)-2 approved by the US Food and Drug Administration in 1992 and, more recently, with programmed cell death protein 1 (PD-1) antibody immune checkpoint blockade (ICB) approved in 2015, followed by cytotoxic T-lymphocyte-associated protein 4 (CTLA-4) and programmed death-ligand 1 antibody ICB.^2^ A tumor-associated antigen (Ag) peptide vaccine, IMA901, was assessed in phase III clinical trials but did not improve patient outcomes.^3^ Within preclinical research, the mouse renal adenocarcinoma RENCA model is a commonly used tumor model to assess RCC immunotherapies. The development of tools for evaluating new immunotherapies based on induction of CD8^+^ cytotoxic T lymphocytes, specifically those that can recognize tumor-specific Ags such as neoepitopes, could be useful to accelerate RCC research.

CD8^+^ T-cell responses form the backbone of cancer immunotherapy and are initiated and executed by forming the peptide–major histocompatibility class I (MHC-I) T-cell receptor (TCR) complex.^4^ These short peptides are 8–10 amino acids in length and need to be expressed, bound, and processed from their parent proteins to be displayed on MHC-I surfaces of antigen-presenting cells (APCs) or target cells, where they would be engaged by a CD8^+^ T cell expressing an appropriate TCR for that neoepitope.^5–8^ CD8^+^ T-cell neoepitopes include tumor-specific, mutated MHC-I binding epitopes and represent a rich source of personalized cancer vaccine targets. In theory, these bypass challenges related to central immune tolerance, and mutations from primary tumors (that would be analyzed to identify neoepitopes) are maintained during disseminated disease.^9^ However, since patients rarely share neoepitopes,^10^ understanding which ones represent functional immunogenic targets has been challenging. Exploratory clinical trials with dendritic cell vaccines using a small cocktail of short peptide neoepitopes have shown that neoepitope-specific CD8^+^ T cells can be induced in humans.^11^ However, induction alone is not sufficient for cancer therapy since, in addition, those epitopes need to be displayed in sufficient quantity in the context of the appropriate MHC-I of the tumor target. As a result of these challenges, namely, predicting which neoepitopes are immunogenic and which give rise to functional CD8^+^ T-cell responses, multivalent immunization strategies are common, with the intent that even a single epitope from a neoepitope cocktail would elicit therapeutic efficacy.

Even though short, synthetic MHC-I epitopes are low-cost, contain the required biochemical information to induce Ag-specific CD8^+^ T cells, and represent the most rational target for injectable neoepitope vaccines, virtually all recent clinical^12–15^ and preclinical^16–19^ neoepitope discoveries make use of long epitope immunogens (typically 25–100 amino acids) that are less relevant for direct or efficient binding to the MHC-I molecule. Indeed, when MHC-I neoepitopes are targeted with long epitope immunogens, off-target binding to MHC-II occurs, resulting in indirect CD4^+^ T-cell responses that drive antitumor immunity.^20^ Thus, while targeting CD4^+^ T-cell epitopes via long immunogens is a dominant strategy that provides help for CD8^+^ T-cell generation,^21^ it confounds direct induction of Ag-specific CD8^+^ T cells to lyse tumor cells from a pool of neoepitope candidates selected on the basis of MHC-I binding.

To make use of short, synthetic MHC-I epitopes, we recently developed the so-called CPQ (CoPoP/PHAD-3D6A/QS-21) liposome adjuvant system that contains (1) cobalt porphyrin–phospholipid (CoPoP) to bind with peptide bearing a polyhistidine-tag (his-tag) with simple mixing, (2) a synthetic Toll-like receptor 4 agonist phosphorylated hexa-acyl disaccharide (PHAD)−3D6A, and (3) the saponin QS-21. CPQ liposomes were recently shown to potently induce Ag-specific CD8^+^ T-cell responses when admixed with nanogram doses of short peptide immunogens in prophylactic and therapeutic tumor models.^22 23^ In the present work, we make use of next-generation sequencing technology with highly multiplexed CPQ particles to identify a short neoepitope capable of cancer ablation in the RENCA mouse model of RCC.

## Materials

Short RENCA neoepitope peptides were synthesized by GenScript. Synthetic peptide information is listed in online supplemental table S1. Control peptides CT1–CT59 were synthesized by Genscript, and those sequences and peptide information are described in a recently published work.^22^ CoPoP was produced as previously described.^24^ The lipids used were: DOPC (Corden, catalog number LP-R4-070), cholesterol (PhytoChol, Wilshire Technologies), and synthetic PHAD-3D6A (Avanti, catalog number 699855). QS-21 was obtained from Desert King (catalog number NC0949192). Polyinosinic:polycytidylic acid (poly (I:C)) was obtained from Sigma (catalog number P1530) and InvivoGen (catalog number 31852-29-6). Anti-mouse CD40 antibody was obtained from BioXcell (clone FGK45, catalog number BE0016-2). Live/dead dye was obtained from Invitrogen (catalog number L34957). Antibodies were obtained from BioLegend: APC-CD8a antibody (catalog number 100712), FITC-CD4 antibody (catalog number 100405), APC-Cy7-CD44 (catalog number 103027), PE/Cy7-CD62L antibody (catalog number 104417), FITC-PD-1 (catalog number 135213), PerCP-Cy5.5-TIM-3 (catalog number 134011), PE-Cy7-LAG3 (catalog number 125225), Alexa Fluor 700-CD45 (catalog number 103127), Brilliant Violet 785-CD3 (catalog number 100231), pacific blue-interferon gamma (IFN-γ) (catalog number 505818), FITC-IFN-γ (catalog number 505805), and BV605-tumor necrosis factor alpha (TNF-α) (catalog number 506329). For checkpoint blockade, anti-mouse PD-1 (clone RMP1-14, catalog number BP0146) and anti-mouse CTLA-4 (clone 9H10, catalog number BP0131) were obtained from BioXCell. Golgiplug (BD catalog number 555029), Fc-block (BD, catalog number 553142), and fixation/permeabilization kit (BD, catalog number 554714) were used for flow cytometry. Cell lysis buffer was from BioVision (catalog number 5830). Collagenase type I was from Gibco (catalog number 17 018–029). DNase I (catalog number 04536282001) was from Roche Diagnostics. ELISpot kits were from Mabtech (catalog number 3321-4ATP-2).

10.1136/jitc-2021-003101.supp1 Supplementary data

## Methods

### Sequencing of RNA from BALB/c mice and RENCA tumor cells

Whole exome sequencing libraries were prepared using the SureSelect*^XT^* Mouse All Exon Kit from Agilent according to the manufacturer instructions. Paired-end sequencing was performed on Illumina NextSeq platform to produce 75 bp reads. For the RNA-Seq experiment, we used the Illumina Stranded TruSeq RNA library preparation kit, followed by 75-cycle paired-end sequencing on the NextSeq in mid-output mode, generating approximately 25 million reads per sample.

To make variant calls from whole exome sequencing data, we first aligned the raw sequencing reads (in fastq format) to the GRCm38 reference assembly using BWA V.0.7.13 (with the ‘mem-M’ option), followed by merging and sorting (by genomic coordinates) of the individual bam files from the same sample sequenced on multiple flow-cell lanes. We then used the Mutect2 tool from the Genome Analysis Toolkit (GATK V.4.0.9.0) with default parameters to make variant calls using the coordinate-sorted bam file from tumor cell line as input for ‘-tumor’ and the bam file from BALB/c as input for ‘-normal’. We then filtered the variants in the resulting variant call format (VCF) file using the ‘FilterMutectCalls’ tool in GATK. The filtered VCF files were further normalized by splitting multiple alleles and left-aligning indels using the bcftool in the samtools suite (http://samtools.github.io/bcftools/bcftools.html). The final VCF file was input to the online Ensembl Variant Effect Predictor (http://useast.ensembl.org/Mus_musculus/Tools/VEP) tool for variant functional effect annotation. For RNA-seq data, we first aligned the raw sequencing reads from the tumor cell lines as well from the BALB/c sample to the GRCm38 reference genome using STAR V.2.6.1b_10–01 with the two-pass approach. The resulting bam files were used to make variant calls using Mutect2. We then filtered the VCF files using the ‘VariantFiltration’ tool in GATK with ‘-window 35 -cluster 3 -filterName FS -filter ‘FS >30.0’ -filterName QD -filter ‘QD <2.0’ as parameters. To get the final candidate non-synonymous variants, we filtered the variants in the exome sequencing VCF file that are annotated as non-synonymous and are also detected in RNA-seq data with a minimum read depth of 4. We then extracted the 9 a.a. peptide sequences affected by these non-synonymous variants sites at different locations (centered around a.a. position 1–9) using a custom Perl script and used the resultant peptide sequences as input for binding affinity prediction using the NetMHC-I binding neural network prediction server (http://www.cbs.dtu.dk/services/NetMHC/).^25 26^

### Vaccine preparation and characterization

Liposomes were formulated using an ethanol injection and lipid extrusion method.^27^ Ethanol was then removed by dialysis in phosphate-buffered saline (PBS) at 4°C, followed by a sterile filtration step using a 0.2 µm sterile filter. For CPQ and 2HPQ preparation, QS-21 (1 mg/mL) was added to liposomes with a DOPC:cholesterol:CoPoP/PoP:PHAD:QS-21 mass ratio of 20.0:5.0:1.0:0.4:0.4. To prepare peptide vaccines, liposomes and peptides were incubated at a mass ratio of 4:1 CoPoP:peptide for 1 hour at room temperature. For desired Ag dosing, liposomes were incubated with Ag then diluted in PBS. To prepare poly(I:C) vaccines, peptides were admixed with poly (I:C) with a dose of poly(I:C) was 50 µg per mouse. To prepare poly(I:C) plus anti-CD40 antibody vaccine, 50 µg peptide was combined with 50 µg anti-CD40 antibody plus 100 µg poly(I:C) (InvivoGen) in PBS. To prepare Alum vaccine, 0.5 or 50.0 µg nesprin-2 L4492R (Nes2LR) peptide was mixed with 2 % aluminum gel (alum; Accurate Chemical and Scientific Corp., catalog number A1090BS) for an hour and diluted with HEPES buffer before injection.

To assess peptide binding to liposomes, peptides were incubated with liposomes or PBS for an hour at room temperature and then a microcentrifugal filtration assay with a 100 kDa cut-off (PALL, catalog number 29300) was used to separate free peptides from liposomes. The concentration of free peptide in the filtrate was determined with micro BCA (Thermo, catalog number 23235). Light scattering with a NanoBrook 90Plus PALS instrument measured the sizes and polydispersity index (PDI) of the samples in PBS and zeta potential in water.

To examine the morphology of CPQ liposomes before and after binding of the Nes2LR peptide, cryoelectron microscopy was used. For sample vitrification, holey carbon grids (C-Flat 2/2-3Cu-T) were first washed in chloroform for 2 hours. Then, sample vitrification was performed using a Vitrobot Mark IV (Thermo Fisher Scientific) with the climate chamber set at 25°C and 100% relative humidity. Before sample application, holey carbon grids were treated with negative glow discharge in air at 5 mA for 15 s. A volume of 3.6 µL of the sample was applied to a holey carbon grid and manually blotted using the Vitrobot blotting paper (Standard Vitrobot filter paper, Ø55/20 mm, grade 595). Right after blotting, a new drop of 3.6 µL of the same sample was applied a second time to the holey carbon grid and blotted once in the Vitrobot for 3 s using a blot force +1. The grid was then plunged into liquid ethane. Samples were imaged at FEMR-McGill with a Tecnai F20 transmission electron microscope operated at 200 kV using a side-entry Gatan 626 single tilt cryoholder. Images were collected in a TVIPS XF416 CMOS camera at a magnification of ×62,000, which produced images with a calibrated pixel size of 1.761 Å. Images were acquired with a total dose of ~ 50 e−/Å2 using a defocus ranging from −1.75 µm to − 2.50 µm.

### Cell studies

RENCA cells were obtained from the American Type Culture Collection and cultured in Roswell Park Memorial Institute (RPMI) 1640 supplemented with 10% fetal bovine serum (FBS), 1 % penicillin-streptomycin (pen/strep), 0.1 mM extra non-essential amino acid, 1 mM extra sodium pyruvate, and 2 mM extra L-glutamine. Mouse kidney carcinoma RENCA^LUC+^ cell expressing luciferase have been described previously.^28 29^ Cells were maintained in RPMI 1640 supplemented with 5% FBS. For the splenocyte studies, spleens were dissociated and filtered through a 70 µm cell strainer. 5 mL of cold PBS was used to wash cells into a 50 mL tube. Cells were centrifuged at 500×*g* for 5 min, the supernatants were discarded. Red blood cells were lysed with 5 mL lysis buffer for 5 min, then 35 mL PBS was added to the tube. Cells were centrifuged again, and cell pellets were collected for further use. For tumor-infiltrating lymphocyte (TIL) studies, tumors were cut into small pieces, then digested with collagenase type I (2 mg/mL) and DNase I (100 μg/mL) for an hour. Cells were passed through a 70 µm strainer, and washed for further experiments. Peripheral blood mononuclear cells (PBMCs) were prepared by lysing 100 µL of whole blood with 2 mL of RBC lysis buffer and washing twice for further use. Splenocytes, TILs, and PBMCs were cultured in RPMI 1640 supplemented with 10% FBS, 1% pen/strep, glutamine (2 mM), sodium pyruvate (1 mM), 1× diluted non-essential amino acid solution, and β-mercapethanol (50 µM). Cells were cultured in 5% CO_2_/95% air at 37°C in a humidified chamber.

### Murine studies

Murine studies were performed according to protocols approved by the University at Buffalo Institutional Animal Care and Use Committee (IACUC). Studies involving orthotopic RENCA^LUC+^ implantation performed at Roswell Park Comprehensive Cancer Center (RPCCC), these studies were approved by the IACUC at Roswell Park according to Protocol 1227M.

5–6 week-old female BALB/c mice (Charles River Laboratory) were immunized intramuscularly with 50 µL vaccine. For the prophylactic vaccine tumor model, mice were vaccinated on days 0 and 7, and challenged with 2×10^5^ RENCA cells subcutaneously on day 14. For the therapeutic vaccine tumor model, BALB/c mice were inoculated with 1×10^5^ tumor cells subcutaneously on the right flank on day 0, and then vaccinated with the indicated vaccine on days 3 and 10 or days 6 and 13 then boosted 7 days after the prime vaccination. For vaccine and anti-PD-1 and anti-CTLA-4 combinational therapy, mice were inoculated with 1×10^5^ tumor cells subcutaneously on the right flank on day 0, and then vaccinated intramuscularly on days 10 and 17. Anti-PD-1 and anti-CTLA-4 (100 µg per antibody per mouse) were administered intraperitoneally on days 12, 14, 19, and 21. Tumor volumes were calculated with the equation: volume=length×width^2^/2. Animals were euthanized after the tumor size reached 1 cm in diameter or when animals developed an ulceration. For the experimental lung metastasis tumor model, animals were injected with 1×10^5^ RENCA cells intravenously via tail vein on day 0, then untreated or intramuscularly injected with the indicated vaccines on days 3 and 10. Lungs were excised and stained with Bouin’s solution (Sigma, catalog number HT10132) on day 23. Tumor nodules were counted, and lung weight was measured. For the orthotopic RENCA^LUC+^ model, luciferase-expressing RENCA^LUC+^ (5×10^4^ cells in 2.5 µL RPMI and 2.5 µL Matrigel) were implanted orthotopically into the left kidney (subcapsular space) of 6–8 week old female BALB/c mice, as described previously.^28–31^ Two and 9 days after tumor inoculation, mice remained untreated or vaccinated with CPQ/Nes2LR with 2 µg of Ag. Animals were monitored with bioluminescence (BL) to quantify tumor burden. Animals were sacrificed by cervical dislocation followed by necropsy within 24 hours when the end-stage disease was reached, which was defined in approved RPCCC IACUC protocols and prior published protocols.^29^

### Antibody staining

For tetramer staining, immunized mice were analyzed for the percentages of tetramer^+^ cells of CD8^+^ T cells by a tetramer staining assay. H-2K^d^-restricted Nes2LR (AYTTQREEL) peptide was complexed with MHC-I (H-2K^d^) and conjugated with PE (the NIH Tetramer core facility). PBMC from 100 µL of blood or 1×10^6^ splenocytes or 5×10^6^ tumor infiltrating cells were incubated with the tetramer (100× dilution) for an hour at 4℃. For T-cell phenotyping, antibody mixtures of Fc-block (100× dilution), live/dead dye (500× dilution), CD8a (200× dilution), CD44 (200× dilution) and CD62L (200× dilution) were added to the cells. For T-cell exhaustion study, antibody mixtures of Fc-block (100× dilution), live/dead dye (500× dilution), CD3 (200× dilution), CD8a (200× dilution), PD-1 (200× dilution), TIM-3 (200× dilution) and Lag-3 (200× dilution) were added to cells. Cells were incubated with these antibodies for 30 min at 4°C, then washed twice for flow cytometry analysis. Flow cytometry was carried out using a BD LSRFortessa X-20 cytometer. FlowJo V.10 software was used for data analysis.

For intracellular staining, PBMCs from 100 µL blood or 1×10^6^ splenocytes or 5×10^6^ tumor infiltrating cells in 100 µL cell culture medium were seeded in a flat bottom 96-well plate and stimulated with Ag (10 µg/mL) for 15–18 hours in the cell culture incubator. GogliPlug (brefeldin A) was added to the plate with a final concentration of 1000× dilution for another 5 hours. Cells were transferred to a 96-well round bottom plate and centrifuged at 1350 rpm. The cell pellet was washed twice and stained with tetramer for an hour at 4℃ then mixed with live/dead dye (500× dilution), Fc-block (100× dilution) and the following antibodies against CD45 (200× dilution) and CD8 (200× dilution) for 25 min at 4°C. Cells were fixed, permeabilized according to the manufacturer’s instruction. Cells were further stained with antibodies against IFN-γ (200× dilution), TNF-α (200× dilution) for 30 min at 4 ℃, then washed for flow cytometry.

For enzyme-linked immune absorbent spot (ELISpot) assay, 3×10^5^ splenocytes or TIL were seeded in an ELISpot plate, and 10 µg/mL peptide was added to each well. Cells were cultured in 5% CO_2_/95% air at 37°C in a humidified chamber for 24 hours. The detection of spots was performed according to the manufacturer instructions. Images were acquired with an Echo REBEL microscopy with a 4× objective and the number of spots was counted with ImageJ software.

For the CTL cytotoxicity assay, isolated splenocytes were cultured in cell culture medium and stimulated with mouse IL-2 (Pepro Tech, catalog number 212–12; 10 IU/mL) and Ags (10 µg/mL) for 5 days to use as the effector cells. Five thousand RENCA cells or irrelevant TC-1 cells, as target cells, were seeded in a 96-well plate and pulsed with Ag (10 µg/mL) or without Ag for an hour at 37℃. Then splenocytes were added to the plate at different effector:target cell ratios for 5 hours. The cytotoxicity of splenocytes on RENCA cells was assessed by lactate dehydrogenase release using a non-radioactive cytotoxicity assay kit (Promega, catalog number G1780) according to manufacturer instructions.

### DNA sequencing of Nes2LR in RENCA cells

DNA was extracted and PCR-amplified using forward primer GCCTTCACTAATTGGGTTTCTCT and reverse primer CCTAAGACTCACTCCATTATGACTC. Purified DNA was sequenced by the Sanger sequencing method at the DNA Sequencing Laboratory (Roswell Park Comprehensive Cancer Center, Buffalo, New York, USA). Data were analyzed with SnapGene software.

### Statistical analysis

Data were analyzed with Prism V.9 (GraphPad Software) using the tests described in the figure captions. P values less than 0.05 were considered statistically significant. Values are generally reported as mean±SD with the indicated sample size.

## Results

### Multivalent immunization with short neoepitope particles

**Figure 1:**
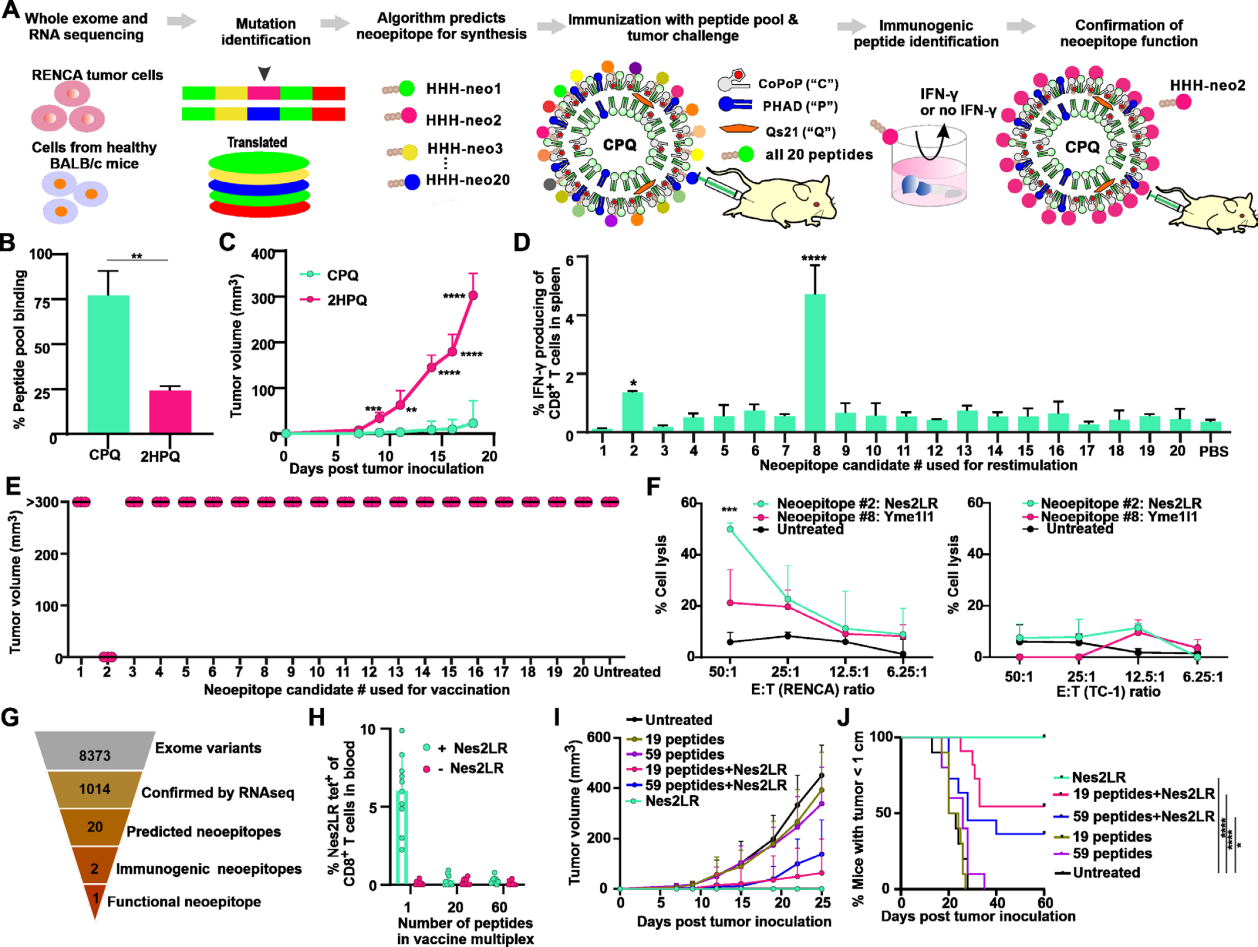
Multivalent immunization reveals Nes2LR as a functional neoepitope. (A) Approach used for in vivo screening of RENCA neoepitopes. (B) Binding synthetic neoepitope candidate pool to CPQ or 2HPQ liposomes. (C) BALB/c mice were immunized with CPQ or 2HPQ liposomes admixed with all 20 predicted neoepitope candidates (50 ng per peptide) on days 0 and 7, then challenged with RENCA cells subcutaneously on day 14, and tumor growth was monitored. (D) Eighteen days after tumor inoculation, spleens were collected from the group immunized with CPQ, and the neoepitope pool, and splenocytes were restimulated with individual neoepitope candidates and assessed for intracellular IFN-γ staining. (E) Mice were immunized with CPQ admixed with individual neoepitope candidates (0.5 µg peptide per mouse) on days 0 and 7, then challenged with RENCA cells on day 14. Tumor sizes on day 21 are shown. (F) Splenocytes from immunized mice were cultured with Ag and interleukin-2 for 5 days before serving as effector cells to lyse tumor target cells at indicated effector to target (E:T) cell ratio. (G) Flowchart of the neoepitope identification and processing during the screening. Mice were vaccinated with CPQ with the Nes2LR neoepitope along with another 19 or 59 unrelated short peptides on days 0 and 7, then challenged with RENCA cells on day 14 with a total Ag dose of 3 µg peptide and assessed for (H) percentage of Nes2LR-tetramer^+^ cells in the CD8^+^ T-cell population, (I) tumor growth and (J) percentage of mice bearing tumors not exceeding 1 cm. Error bars show mean±SD for n=3 or four replicates (B, F, respectively), or n=5 mice (C, D) or n=3 mice (E) or n=10 mice (H–J). *P<0.05, **P<0.01, ***P<0.001, ****P<0.0001, analyzed by the (B, C) two-tailed unpaired Student’s t-test or (D, F) one-way analysis of variance with Dunnett’s multiple comparisons post-test or (J) log-rank test. IFN-γ, interferon gamma; Nes2LR, nesprin-2 L4492R; RENCA, renal adenocarcinoma.

The overall approach of multivalent immunization is outlined in figure 1A. Neoepitope candidates are identified with genomic approaches, ranked based on MHC-I binding, synthesized, and then immunized in the form of highly multiplexed immunogenic short peptide particles. Following assessment of antitumor activity, individual neoepitopes are assessed and further analyzed to identify which ones are stimulatory.

Whole exome RNA and DNA sequencing was carried out using RENCA murine RCC cells, along with control tissue from parental BALB/c mice, to identify mutations in the tumor cells. A neural network algorithm was used to rank all non-synonymous 9-mer neoepitopes within the top 0.1 percentile for binding affinity for all 3 BALB/c MHC-I haplotypes, and 20 neoepitope candidates were identified, which were then chemically synthesized with conventional solid-phase peptide chemistry (online supplemental table S1). Three histidine residues were included on the N-terminus to enable particle formation with CPQ liposomes. Incubation of the peptide pool with the liposomes resulted in the binding of approximately 80% of the total peptide pool, while liposomes lacking cobalt (‘2HPQ’ liposomes, that substitute two protons for cobalt) had diminished binding (figure 1B). There was no significant change in liposome size with or without peptide binding, which remained ~100 nm (online supplemental figure S1A). The PDI of liposomes was less than 0.25 (online supplemental figure S1B), reflecting a reasonably monodisperse population.

First, we sought to investigate antitumor efficacy of the multiplexed peptides in a prophylactic setting. To this end, BALB/c mice were vaccinated intramuscularly with the multivalent peptide particles on days 0 and 7 with a dose of 50 ng of each peptide (1 µg total peptide dose) and then were challenged with RENCA cells subcutaneously on day 14. Mice immunized with the multivalent particle vaccine had significantly smaller tumor sizes compared with the 2HPQ control liposomes, which contained identical composition except they lacked cobalt so that the neoepitopes did not form stable particles (figure 1C). Splenocytes were collected from mice 18 days after tumor implantation and were restimulated with 10 µg/mL of each peptide individually. Only the Nes2LR and Yme1L1A539G short neoepitopes induced elevated percentages of IFN-γ producing CD8^+^ T cells, suggesting they induced Ag-specific CD8^+^ T cells as a result of the multivalent immunization (figure 1D).

To determine which neoepitopes were responsible for the antitumor efficacy of the multiplexed particle vaccine, each was tested individually for tumor growth inhibition following immunization. Of all 20 neoepitope candidates, only Nes2LR inhibited tumor growth and did so completely (figure 1E). Eighteen days after tumor inoculation, mice were sacrificed, and splenocytes were prepared and restimulated with neoepitope Ag and IL-2 to confirm the generation of cytotoxic effector cells. Indeed, cytotoxicity studies revealed splenocytes from mice immunized with CPQ/Nes2LR, but not CPQ/Yme1L1A539G, could specifically lyse RENCA cells but not irrelevant TC-1 cells (figure 1F). The lack of antitumor efficacy of Yme1L1A539G may be due to insufficient epitope expression on MHC-I on the tumor cell surface. Thus, the paradigm to identify short neoepitopes resulted in 8373 mutations found in the exome of RENCA cells and 1014 non-synonymous coding variants (figure 1G). Of the 20 neoepitopes with MHC-I binding in the top 0.1 percentile, only two induced Ag-specific CD8^+^ T cells as detected by IFN-γ production, and just one induced an antitumor response.

Considering the low frequency of functional neoepitopes, multiplexing strategies are of particular importance for related vaccine approaches.^12 15^ To assess how effectively multivalent immunization with CPQ would present neoepitopes and induce Ag-specific CD8^+^ T cells in vivo, the Nes2LR neoepitope was combined with 19 or 59 other irrelevant neoepitopes from the CT26 murine cancer cell line (peptide CT1-CT59, described in our recently published work^22^). Peptide particles were formed by simple admixing with CPQ liposomes, and the dose for each peptide, including Nes2LR, was 150 ng for the 20-plex particle vaccine and 50 ng for the 60-plex particle vaccine, for a total peptide dose of 3 µg. While 3 µg of monovalent CPQ/Nes2LR induced ~6% of CD8^+^ T cells in blood to become Nes2LR-tetramer (tet)^+^, the 20 and 60 plex did not yield detectable Nes2LR-tet^+^ CD8^+^ T-cell populations (figure 1H). Nevertheless, we challenged mice with tumor cells to assess the antitumor efficacy of these multiplexed vaccines. CPQ/Nes2LR, as well as CPQ/20-plex and CPQ/60-plex, all substantially inhibited tumor growth (figure 1I) and increased the percentage of mice with tumor sizes smaller than 1 cm (figure 1J) in a prophylactic setting. Thus, a small dose of the Nes2LR epitope even in a background of a highly multivalent CPQ particle vaccine was of sufficient immunogenicity to provide for functional antitumor efficacy.

**Figure 2:**
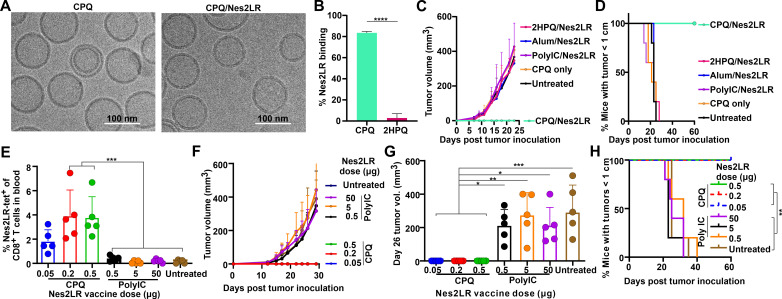
Antitumor effects of Nes2LR neoepitope immunization when converted into particle format by admixture with CPQ liposomes. (A) Cryo-TEM images of CPQ liposomes with or without Nes2LR peptide bound. (B) Binding percentage of Nes2LR peptide to CPQ and 2HPQ liposomes. BALB/c mice were vaccinated on days 0 and 7, and then blood was collected for flow cytometry analysis on day14; mice were challenged with RENCA tumor cells subcutaneously on day 14. (C) Tumor volume of mice. (D) Mice with tumor sizes smaller than 1 cm. (E) Percentage of Nes2LR-tet^+^ cells in the CD8^+^ cell population in blood. (F) Tumor growth of mice. (G) Tumor volume of mice on day 26. (H) Percentage of mice with tumor sizes smaller than 1 cm. Error bars show mean±SD for n=5 mice. *P<0.05, **P<0.01, ***P<0.001, ****P<0.0001, analyzed by (B) two-tailed unpaired Student’s t-test or (E, G) one-way analysis of variance with Bonferroni multiple comparisons post-test or (H) log-rank test. Nes2LR, nesprin-2 L4492R; RENCA, renal adenocarcinoma.

Properties of CPQ/Nes2LR particles and their immunogenicity were further investigated. The DNA mutation responsible for the Nes2LR neoepitope was confirmed in RENCA cells by Sanger sequencing of genomic DNA and presented itself as a heterozygous mutation (online supplemental figure S2). Based on cryo-transmission electron microscopy (cryo-TEM), the morphology of CPQ liposomes remained spherical with liposome sizes ~100 nm with or without Nes2LR bound (figure 2A). Dynamic light scattering confirmed the cryo-TEM observations, with sizes ~100 nm with or without peptide binding (online supplemental figure S3A) and PDI less than 0.2 (online supplemental figure S3B). Admixing the Nes2LR peptide with CPQ liposomes resulted in the conversion of approximately 80% of the peptide into particle format, whereas a negligible amount of peptide bound to 2HPQ liposomes lacking cobalt (figure 2B).

BALB/c mice were immunized on days 0 and 7 with 0.5 µg Nes2LR. Based on immune analysis of splenocytes, when Nes2LR was converted into particle format, vaccinated mice had a significantly higher percentage of Nes2LR-tet^+^ cells (online supplemental figure S4A), central memory T cells (T_CM_, CD44^+^CD62L^+^) (online supplemental figure S4B), and effector memory T cells (T_EM_, CD44^+^CD62L^-^) (online supplemental figure S4C) in the CD8^+^ T-cell population compared with 2HPQ/Nes2LR vaccinated mice and untreated mice. Mice vaccinated with CPQ/Nes2LR produced a higher percentage of IFN-γ^+^-producing and TNF-α^+^-producing CD8^+^ T cells after Nes2LR restimulation in vitro (online supplemental figure S4D, E). This shows that particle formation of Nes2LR was essential for inducing a robust cellular immune response in mice.

Next, 0.5 µg of Nes2LR was used to immunize mice following admixture with CPQ, 2HPQ, or the common adjuvants, poly(I:C) and alum. Only the CPQ/Nes2LR vaccine resulted in the rejection of the RENCA tumor challenge (figure 2C) and conferred a higher percentage of mice that maintained tumor sizes smaller than 1 cm (figure 2D). Since poly(I:C) is considered a potent vaccine adjuvant and has been used in Ag screening with peptide doses of 50 µg,^17^ we compared the CPQ adjuvant system with poly(I:C) with different Ag dosing. CPQ liposomes were admixed with 0.05, 0.2, or 0.5 µg of peptide, and poly(I:C) was admixed with 0.5, 5.0 and 50.0 µg of peptide. The CPQ dose was set at a fixed ratio with the peptide dose (so that the 0.05 µg group included 0.08 µg both of QS-21 and PHAD-3D6A), whereas 50 µg poly(I:C) adjuvant was used in all cases. Following immunization, even the lowest dosage of CPQ/Nes2LR induced higher frequencies of Nes2LR-tet^+^ CD8^+^ T cells compared with the same peptide immunogen dosed at 1000 times greater level but admixed with poly(I:C) (figure 2E). Immunization with CPQ/Nes2LR completely inhibited tumor growth (figure 2F), with no tumor growth detected ~30 days after challenge (figure 2G), whereas control mice and mice treated with poly(I:C)/Nes2LR rapidly developed tumors greater than 1 cm in length (figure 2H). We compared immunization with 0.5 µg Nes2LR admixed with CPQ to 50 µg peptide admixed with alum, or to 50 µg peptide admixed with poly(I:C) administered with anti-mouse CD40 antibody. Vaccination with CPQ produced greater numbers of Nes2LR-tet^+^ T cells in the CD8^+^ T-cell population in the blood compared with any other group (online supplemental figure S5A, B). Upon Ag stimulation in vitro, substantially higher frequencies of Ag-specific, IFN-γ-secreting CD8^+^ T cells were identified in the spleen and blood from mice treated with CPQ vaccination (online supplemental figure S5C, D).

**Figure 3:**
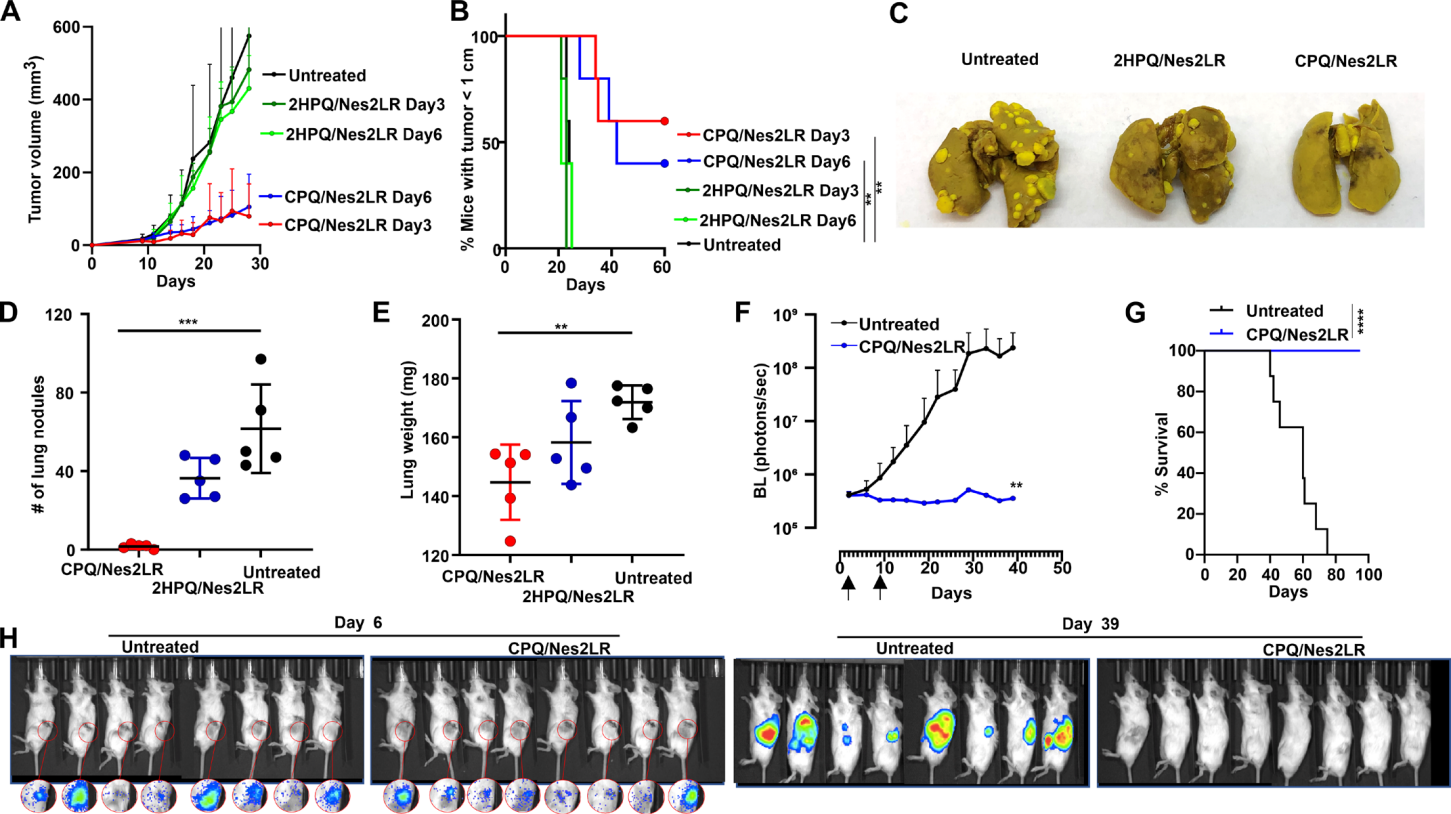
CPQ/Nes2LR as a therapeutic vaccine delayed ectopic and orthotopic primary RENCA tumor growth and lung metastasis. BALB/c mice were inoculated with RENCA cells subcutaneously on day 0 and then primed on day 3 or 6 and boosted 7 days later; the dose of Ag was 0.5 µg per mouse. Tumor growth (A) and survival (B) of untreated mice or mice vaccinated with CPQ/Nes2LR and 2HPQ/Nes2LR. Error bars show mean±SD for n=5 mice per group. BALB/c mice were injected with RENCA cells intravenously on day 0, then the vaccine with an Ag dose of 0.5 µg per mouse was applied on days 3 and 10. Mice were sacrificed on day 23, and lungs were excised and stained with Bouin’s solution. (C) Image of lungs from mice that received the indicated vaccine. (D) Lung nodule counts. (E) Lung weights. Error bars show mean±SD for n=5 mice per group. BALB/c mice were inoculated with mouse RENCA^LUC+^ tumors orthotopically on day 0 and CPQ/Nes2LR vaccine with 2 µg Ag on days 2 and 9. BL quantification (F), survival (G), and corresponding BL imaging (H) of untreated mice or mice vaccinated with CPQ/Nes2LR. Overall survival based on Kaplan-Meier analysis. Error bars show mean±SD for n=8 mice per group. **P<0.01, ***P<0.001, ****P<0.0001, analyzed by long rank test (B, G) or one-way analysis of variance with Dunnett’s multiple comparisons post-test (D, E) and two-tailed unpaired Student’s t-test on day 39 (F). BL, bioluminescence; Nes2LR, Nesprin-2 L4492R; RENCA, renal adenocarcinoma.

As immunization with CPQ/Nes2LR resulted in complete rejection of the RENCA tumor challenge, we extended the analysis of this neoepitope to therapeutic tumor settings. To test how vaccination could delay tumor growth in established subcutaneous tumor models, mice were inoculated with RENCA cells on day 0 and vaccinated on days 3 and 10, or days 6 and 13. Mice vaccinated with CPQ/Nes2LR (0.5 µg peptide) had significantly smaller tumor sizes than mice vaccinated with non-particle-forming 2HPQ/Nes2LR (figure 3A). By 60 days, 40% and 60% of mice had tumor sizes smaller than 1 cm when immunized with CPQ/Nes2LR on days 6 and 3 after tumor inoculation, respectively. Control mice and 2HPQ immunized mice all had to be sacrificed by day 21 (figure 3B).

To evaluate the vaccine efficacy in an experimental metastatic setting, BALB/c mice were intravenously injected with RENCA tumor cells on day 0 and then immunized intramuscularly with CPQ/Nes2LR or 2HPQ/Nes2LR (0.5 µg peptide) on days 3 and 10. Lungs were collected on day 23, and nodules were quantified. Although untreated mice or mice receiving the 2HPQ/Nes2LR vaccine developed numerous lung metastases, we did not observe obvious lung nodules in mice with CPQ/Nes2LR immunization (figure 3C). Untreated mice or mice injected with 2HPQ/Nes2LR had more than 40 lung nodules per mouse (figure 3D). Increased lung weight correlated with lung metastasis, and mice without treatment or immunized with non-particle forming 2HPQ/Nes2LR had heavier lungs than mice immunized with CPQ/Nes2LR (figure 3E).

The antitumor efficacy of the CPQ/Nes2LR vaccine was assessed in an orthotopic RENCA model. BALB/c mice were implanted with luciferase-expressing RENCA (RENCA^LUC+^) cells orthotopically into the left kidney on day 0, then remained untreated or vaccinated with CPQ/Nes2LR on days 2 and 9. To monitor tumor progression, mice were also monitored two times per week for BL to quantify tumor burden. Untreated mice had increased luciferase signal from day 0 to day 39. At the same time, the CPQ/Nes2LR vaccinated mice showed no increase in luciferase signal from day 0 to day 39 (online supplemental figure S6), resulting in a significant difference in BL signal between the untreated group and vaccine group on day 39 (figure 3F). The CPQ/Nes2LR vaccine markedly prolonged survival compared with the untreated group (figure 3G). Taken together, these findings revealed curative antitumor efficacy of the CPQ/Nes2LR vaccine against orthotopic primary tumor growth and distant metastases. We also rechallenged mice from the CPQ/Nes2LR group with RENCA tumor cells subcutaneously 123 days after the initial tumor implantation. All the immunized mice rejected the tumor rechallenge (online supplemental table S2). These results demonstrate the establishment of durable immunological memory after therapeutic vaccination with CPQ/Nes2LR.

**Figure 4:**
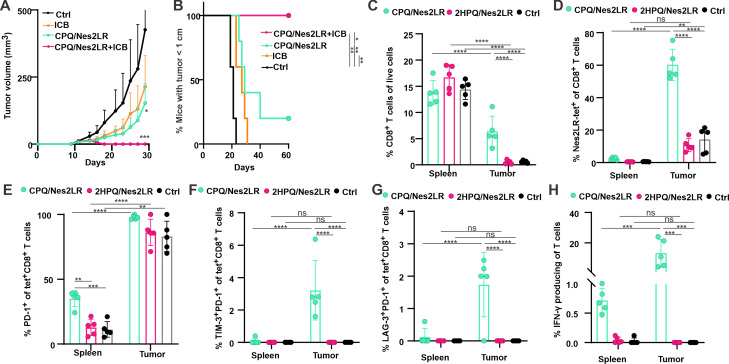
Synergistic effects of CPQ/Nes2LR vaccine and immune checkpoint blockade combination therapy. BALB/c mice were inoculated with tumor cells subcutaneously on day 0 and untreated (Ctrl) or treated with CPQ/Nes2LR on days 10 and 17, treated with ICB (anti-CTLA-4 and anti-PD-1 antibodies on days 12, 14, 19, and 21) or treated with the combination. (A) Tumor growth of mice. (B) Mice with tumor sizes smaller than 1 cm. Error bars show mean±SD for n=5 mice per group. BALB/c mice were inoculated with RENCA tumor cells subcutaneously on day 0 and then untreated (Ctrl) or vaccinated with CPQ/Nes2LR, 2HPQ/Nes2LR on days 10 and 17. Mice were sacrificed on day 22; spleens and tumors were collected for analysis. (C) Percentage of CD8^+^ T cells in the live cells. (D) Percentage of Nes2LR tet^+^ cells in the CD8^+^ T-cell population. Percentage of PD-1^+^ (E), TIM-3^+^ PD-1^+^, (F) and LAG-3^+^ PD-1^+^ (G) among Nes2LR tet^+^CD8^+^ T-cell population. (H) Percentage of IFN-γ producing cells in the CD8^+^ T-cell population after 10 µg/mL Ag stimulation in vitro. Error bars show mean±SD for n=5 mice per group. *P<0.05, **P<0.01, ***P<0.001, ****P<0.0001, analyzed by (A) one-way ANOVA with Dunnett’s multiple comparisons post-test or long rank test (B) or two-way ANOVA (C–H) with Tukey multiple comparisons post-test. The asterisk in A shows a comparison to untreated group on day 29. ANOVA, analysis of variance; CTLA-4, cytotoxic T-lymphocyte-associated protein 4; Ctrl, control; ICB, immune checkpoint blockade; IFN-γ, interferon gamma; Nes2LR, Nesprin-2 L4492R; ns, not significant; PD-1, programmed cell death protein 1; RENCA, renal adenocarcinoma.

Next, we examined the potential synergistic antitumor efficacy of vaccine and ICB using antibodies targeting immune checkpoint receptors. Since the combination of anti-PD-1 and anti-CTLA-4 antibody treatment has been shown to be more effective than anti-PD-1 alone treatment in the RENCA tumor model,^32^ we combined both anti-CTLA-4 and anti-PD-1 antibodies with the vaccine. BALB/c mice were inoculated with tumor cells on day 0 and then were either untreated or immunized with vaccine alone, treated with ICB alone, or treated with the combination of vaccine and ICB. Although ICB alone and CPQ/Nes2LR vaccination alone slowed tumor growth and improved survival compared with the untreated group, a combination of ICB and neoepitope vaccine mediated complete regression of established tumors (figure 4A, B).

We also investigated neoepitope-specific CD8^+^ T cells in the tumor microenvironment (TME), as well as the effect of ICB. BALB/c mice were inoculated with tumor cells on day 0 and then were untreated or vaccinated with CPQ/Nes2LR or 2HPQ/Nes2LR on days 10 and 17. CPQ/Nes2LR vaccination markedly increased intratumoral CD8^+^ T cells containing approximately 60% of Nes2LR-tet^+^ cells, while the frequency of splenic CD8^+^ T cells remained unchanged. In contrast, the untreated and 2HPQ/Nes2LR groups had minimal frequencies of tumor-infiltrating CD8^+^ T cells comprising ~10% of tet^+^ cells. (figure 4C, D, online supplemental figures S7 and S8)

To uncover evidence for T-cell exhaustion within the TME, which could limit therapeutic efficacy, we also assessed the expression of PD-1, T-cell immunoglobulin mucin-3 (TIM-3) and lymphocyte-activation gene 3 (LAG-3). Flow cytometry analyses showed that a higher percentage of Nes2LR-tet^+^ CD8^+^ T cells expressed PD-1 in the TME compared with CD8^+^ T cells in the spleen in both untreated mice and mice vaccinated with CPQ/Nes2LR or 2HPQ/Nes2LR groups (figure 4E). In comparison, mice vaccinated with CPQ/Nes2LR also had a higher percentage of Nes2LR-tet^+^ CD8^+^ T cells that expressed both TIM-3 and PD-1 (figure 4F) or LAG-3 and PD-1 (figure 4G) in the TME compared with the spleen. In the CD8^+^ T cells induced by CPQ/Nes2LR in the TME, a much higher percentage of those T cells expressed PD-1, TIM-3, and LAG-3 in the Nes2LR-tet^+^ T cells compared with Nes2LR-tet^−^ T cells (online supplemental figure S9). We also stimulated splenocytes and tumor-infiltrating cells with peptide Ags in vitro and determined a markedly higher frequency of IFN-γ^+^ cells in the CD8^+^ T-cell population in response to Nes2LR in the spleen and tumor from mice treated with CPQ/Nes2LR vaccination compared with untreated and 2HPQ/Nes2LR vaccinated mice (online supplemental figure S10 and figure 4H). Of note, the T cells induced by CPQ/Nes2LR vaccination were specific to the Nes2LR AYTTQREEL neoepitope and not the parental non-mutated AYTTQLEEL sequence (online supplemental figure S11).

## Discussion

Nanoparticles have reached clinical testing for cancer vaccines based on liposomes,^33–35^ virus-like nanoparticles^36^ and nanogels.^37^ The CPQ adjuvant system, which is based on CoPoP liposome technology that enables spontaneous self-assembly of Ags, has previously been demonstrated to enhance the immunogenicity of soluble protein Ags derived from microbial pathogens,^38–40^ CoPoP liposomes recently have proceeded into human clinical trials (clinicaltrials.gov # NCT04783311) for a SARS-CoV-2 vaccine. The recent demonstration of the CPQ system for cancer vaccines shows a potential role in inducing Ag-specific CD8^+^ T cells using simple, short peptides,^22 23^ and the present work extends this approach to highly multiplexed neoepitopes. Short peptides also offer substantial simplicity in terms of easy production and characterization relative to proteins or even long peptides.

Most neoepitope vaccine approaches are considered in the context of a multiplexed vaccine, and clinical studies typically involve between 6 and 20 immunogens to maximize the potential for immunogenic epitope discovery.^11–14^ However, within preclinical research, similar multiplexing is rarely demonstrated, and immunization during the screening of epitopes typically proceeds only one to two peptides at a time.^17 20^ CPQ appears to be a potent adjuvant system, as we demonstrated that immunization with the single functional Nes2LR neoepitope, codelivered within a cocktail of 60 peptides (the other 59 being non-functional), inhibited the growth of RENCA tumors. However, within this context, the multivalent vaccine appeared to produce a lower frequency of Ag-specific CD8^+^ T cells than the corresponding monovalent vaccine at the same Ag dose. The immunological reasons for this effect could potentially relate to competition for MHC-I binding sites or other interference effects. The upper limit of the number of peptides possible in a functional multiplexed CPQ vaccine has not yet been ascertained. It could be assessed in future work by increasing the multiplex size, along with further optimization of peptide and adjuvant dose.

Even though neoepitopes have attracted broad interest as a potential component of cancer immunotherapy,^41^ very few short functional CD8^+^ T-cell neoepitopes have been identified in mice so far. The Adpgk neoepitope was identified as a CD8^+^ T-cell vaccine Ag against MC38 tumors,^17^ but within the context of longer peptides that also activate MHC-II epitopes. In a separate study, it was assessed as a component of a short peptide delivery system based on particle formation.^42^ The CPQ system can be used for screening to identify neoepitopes in other murine cell lines and in the future in human cancer cell lines using humanized mice that express transgenic human leukocyte antigen and engrafted with human CD8^+^ T cells. We expect the Nes2LR neoepitope identified in the current study could become useful for inducing cytotoxic CD8^+^ T cells in immunotherapy studies for RCC research.

Further dose-dependent toxicity and functional studies are warranted for the CPQ multivalent vaccine system, which could also define the toxic and immunological limits of the highly multivalent approach. We did not observe in vivo toxicities in the current study, in line with our recent preclinical study, which showed that CPQ liposomes are well tolerated in mice.^22^ Based on the synergistic effect of CPQ/Nes2LR vaccination and ICB, further optimization could lead to using neoepitope vaccines to reverse the growth of larger established tumors.

In the current study, we identified more than 1000 protein mutations expressed in the RENCA cell line and tested the 20 associated neoepitopes with the best-predicted MHCI-I binding. Of those, two peptides were found to be immunogenic and just one had functional activity. The ratio of immunogenic MHC-I restricted peptides in the neoepitopes that were screened was ~10%, which is consistent with our previous study.^22^ However, as this percentage is relatively low, computational algorithms that predict immunogenic epitopes could reduce the number of peptides to be tested in screening experiments. To increase the success rate of immunogenic peptide selection, the number and dose of peptides to be included in the multivalent vaccine should be further studied. Using Nes2LR as an immunogen, admixture of the peptide with CPQ induced a more robust functional CD8^+^ T-cell response compared with alum or poly(I: C) combined with anti-CD40 antibody, even at 100-fold and 1000-fold lower peptide dosing. Previous studies have also shown that with the HPV16 E7_49-57_ epitope, CPQ induces a much stronger CD8^+^ T-cell response compared with poly(I: C) or alum even with a 1000 lower Ag dose.^23^ In the current study, we tested the CPQ/Nes2LR vaccine as a therapeutic vaccine in tumors that were established for 2–10 days, but in future work, vaccine efficacy could be tested in larger, more established tumors. Such a setting could be well suited for further development of combination immunotherapies that include neo-Ag targeting components.

## Conclusion

Current cancer neoepitope vaccines based on peptides or mRNA typically rely on long immunogens that usually do not directly induce neoepitope-specific CD8^+^ T cells. Here, we demonstrate this possibility by using multivalent immunization with short MHC-I restricted neoepitope candidates. An effective neoepitope, Nes2LR, was identified following next-generation sequencing of RENCA tumor cells, epitope prediction, and multivalent immunization with a pool of neoepitope candidates. The particle-inducing CPQ adjuvant system was critical for directly inducing neoepitope-specific CD8^+^ T cells. In contrast, a traditional adjuvant such as poly(I:C) was insufficient to be effective with the neoepitope even when peptide dosing was 1000-fold greater. Furthermore, Nes2LR immunization elicited potent antitumor immune responses in prophylactic and therapeutic settings against orthotopic, metastatic, as well as primary tumors and synergized with ICB in a mouse model of kidney cancer. Together, these results suggest that short peptide immunization can be a viable therapeutic approach via multivalent particle immunization, as well as a tool for tumor neoepitope discovery and research of combination cancer immunotherapies.

10.1136/jitc-2021-003101.supp2 Supplementary data

## Data Availability

Data are available upon reasonable request.
