# Supplementary material for: Immunization with short peptide particles reveals a functional CD8+ T-cell neoepitope in a murine renal carcinoma model
Source: J Immunother Cancer. 2021 Dec 3;9(12):e003101. doi: 10.1136/jitc-2021-003101 (PMC8647534; doi:10.1136/jitc-2021-003101)
Supplement: Supplementary data [file jitc-2021-003101supp001.pdf]

Supporting Information: Immunization with Short Peptide Particles Reveals a Functional CD8+ T-Cell Neoepitope in a Murine Renal Carcinoma Model

*Xuedan He[1+], Shiqi Zhou[1+], Melissa Dolan[2], Yuhao Shi[2], Jianxin Wang[3], Breandan Quinn[1], Dushyant Jahagirdar[4], Wei-Chiao Huang[1], Moriya Tsuji[5], Roberto Pili[6], Fumito Ito[7], Joaquin Ortega[4], Scott I. Abrams[7], John M. L. Ebos,[2] Jonathan F. Lovell[1]**

[1.] Department of Biomedical Engineering, University at Buffalo, State University of New York, Buffalo, NY, 14260, USA
[2.] Department of Experimental Therapeutics, Roswell Park Comprehensive Cancer Center, Buffalo, New York, 14263, USA
[3.] Center for Computational Research, University at Buffalo, Buffalo, NY, 14203, USA.
[4.] Department of Anatomy and Cell Biology, McGill University Montreal, Quebec, H3A0C7, Canada
[5.] Department of Medicine, Columbia University Irving Medical Center, New York, NY, 10032, USA
[6.] Department of Medicine, State University of New York, Buffalo, NY, 14203, USA
[7.] Department of Immunology, Roswell Park Comprehensive Cancer Center, Buffalo, NY, 14263, USA

[+] Equal contribution
*E-mail: jflovell@buffalo.edu

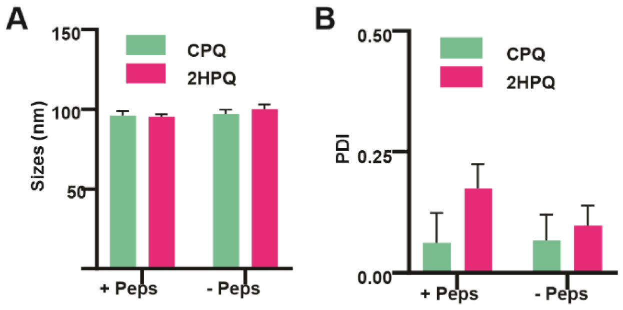

Figure S1. Particle formation with 20 predicted RENCA neoepitope peptides following incubation with CPQ or 2HPQ (control) liposome. Sizes (**A**) and polydispersity (**B**) of liposomes with or without peptide incubation. Error bars show mean +/−std. dev. for n=3 triplicate experiments.

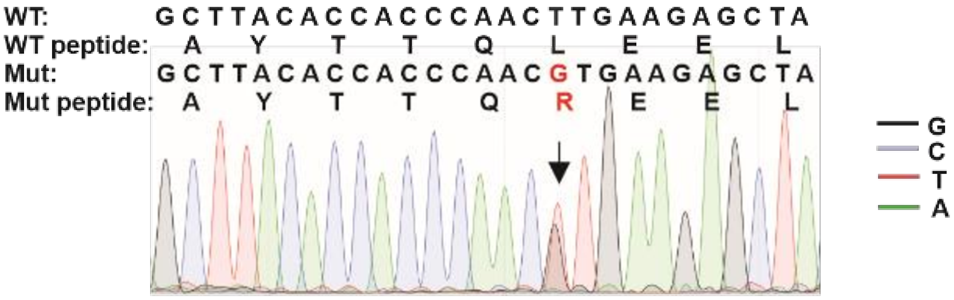

Figure S2. Confirmation of Nes2LR peptide in the RENCA cells. DNA was extracted from RENCA tumor cells and subjected to Sanger DNA sequencing.

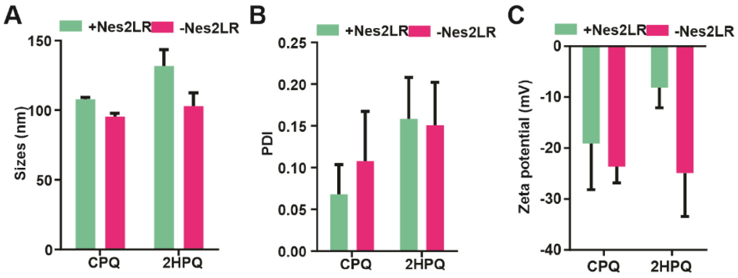

Figure S3. Nes2LR peptide particleized with CPQ liposome. Sizes (**A**), polydispersity (**B**) and zeta potential (**C**) of liposomes with or without peptide bound. Error bars show mean +/−std. dev. for n=3 triplicate experiments.

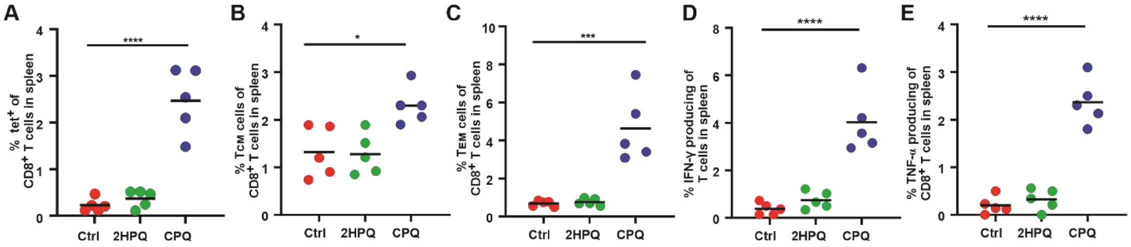

Figure S4. CPQ/Nes2LR vaccine induced strong immune response in the healthy mice. BALB/c mice were either untreated or vaccinated on day 0 and 7, spleens were collected on day 14 for analysis. Percentage of Nes2LR tetramer^{+} cells (**A**), central memory T cells (T_{CM}) ($CD44^{+}CD62L^{+}$) (**B**) and effector memory T cells (T_{EM}) ($CD44^{+}CD62L^{-}$) (**C**) in the $CD8^{+}$ T cell population. Splenocytes were stimulated with 10 µg/mL antigens and percentage of IFN-γ (**D**) and TNF-α (**E**) producing cells in $CD8^{+}$ T cells were analyzed. Error bars show mean +/−std. dev. for n=5 mice. * $p < 0.05$, *** $p < 0.001$, and **** $p < 0.0001$, analyzed by the one-way ANOVA with Dunnett's multiple comparisons post-test.

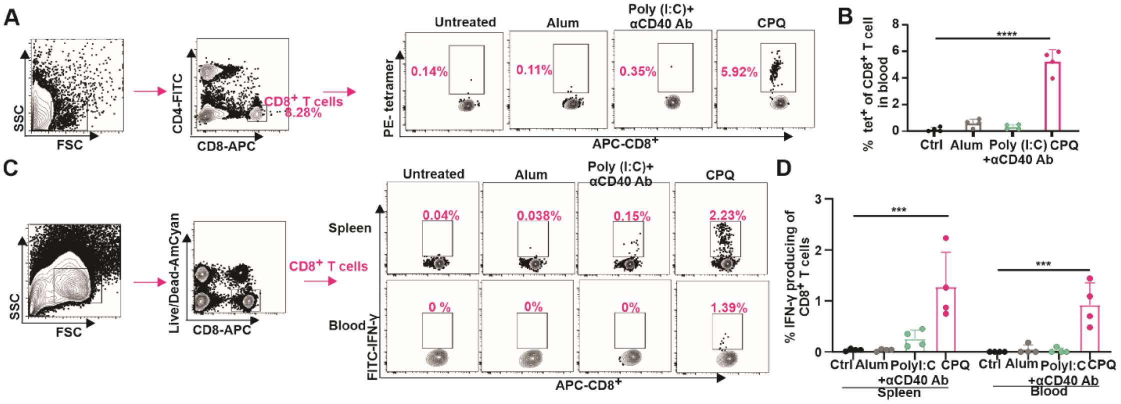

Figure S5. Comparison of immunogenicity of Nes2LR with different adjuvants. BALB/c mice were untreated or injected subcutaneously with 50 µg peptide in combination with 1mg Alum or injected intraperitoneally with 50 µg peptide in combination with 50 µg anti-CD40 (BioXcell, *clone FGK45)* plus 100 µg poly(I:C) (InvivoGen) in PBS, or injected intramuscularly with 0.5 µg peptide in combination CPQ liposome on days 0 and 7, blood and spleen were collected for tetramer and intracellular staining on day 14. Flow cytometry gating (**A**) and percentage (**B**) of Nes2LR tetramer$^+$ cells in the CD8$^+$ T cell population in blood. Flow cytometry gating (**C**) and percentage (**D**) of IFN-γ$^+$ cells in the CD8$^+$ T cell population. Error bars show mean +/−std. dev. for n=4 mice per group. *** $p < 0.001$, and **** $p < 0.0001$, analyzed by one-way ANOVA with Dunnett's multiple comparisons post-test.

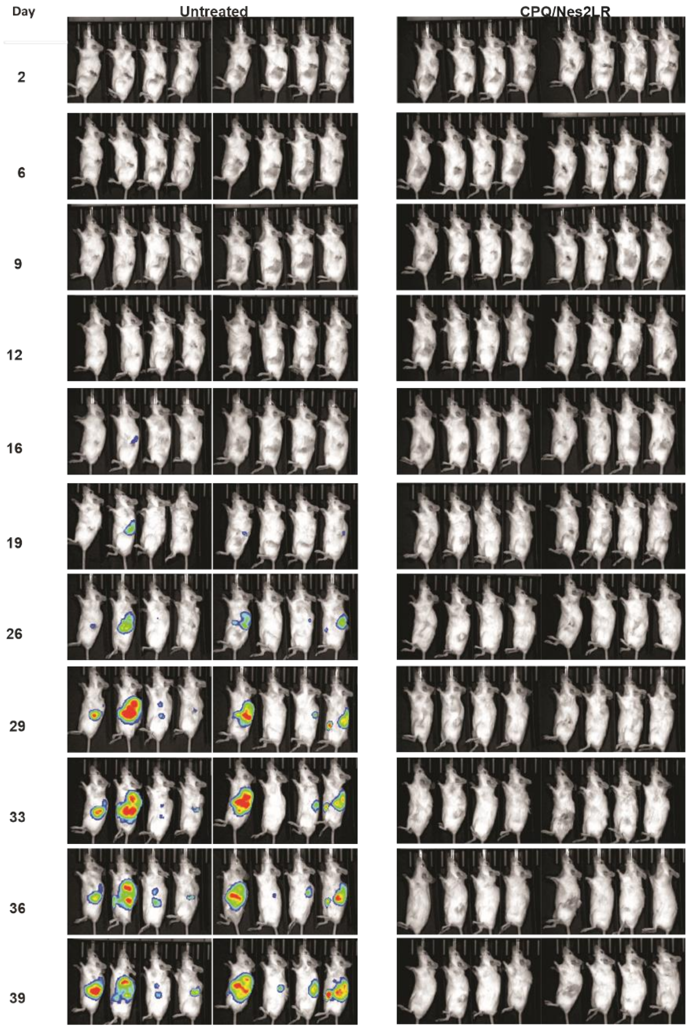

Figure S6. **CPQ/Nes2LR as therapeutic vaccine inhibited tumor growth in orthotopic tumor model.** BALB/c mice were inoculated with mouse RENCA^{LUC+} renal tumors orthotopically on day 0 and CPQ/Nes2LR vaccine with 2 μg antigen per mouse were give on day 2 and 9. Images show bioluminescence (BL) of mice.

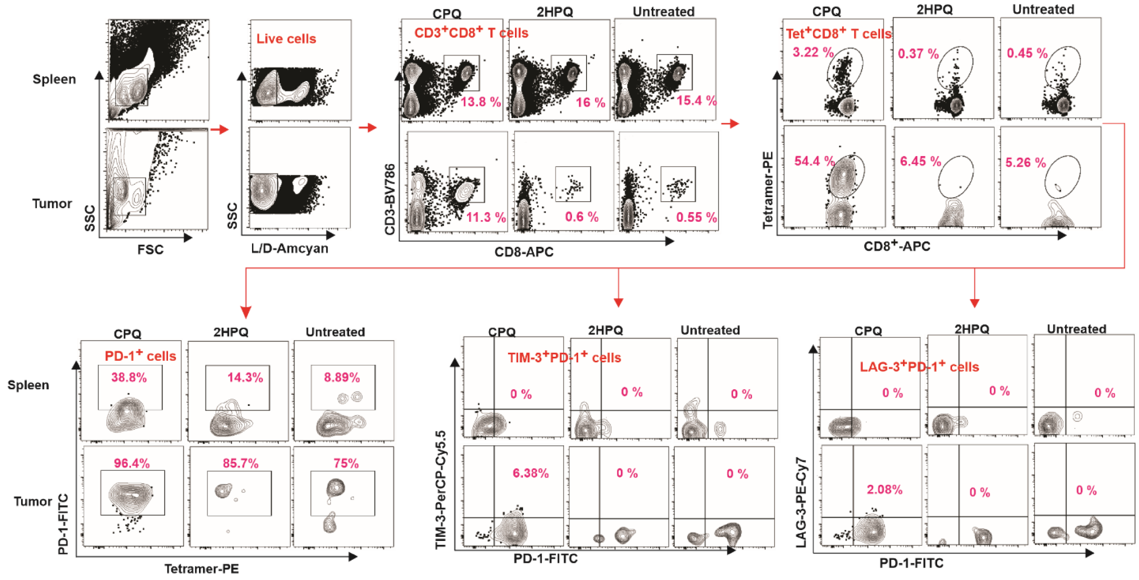

Figure S7. Flow cytometry gating of CD8⁺ T cells in live cells, tetramer⁺ cells in CD8⁺ T cells, PD-1⁺ cells, TIM-3⁺PD-1⁺ and LAG3⁺ PD-1⁺ T cells in tet⁺ T cells.

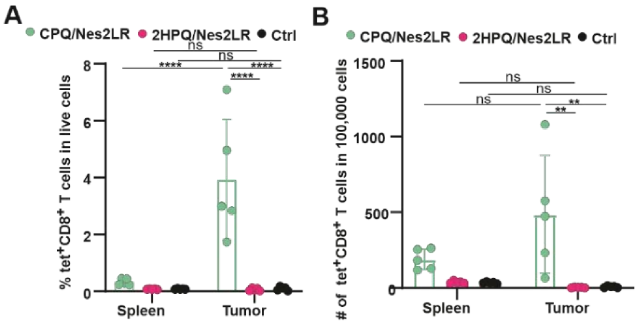

Figure S8. CPQ/Nes2LR vaccination elicited much higher number of tet⁺CD8⁺ T cells in spleen and tumor compared to 2HPQ/Nes2LR vaccination and untreated group. BALB/c mice were inoculated with RENCA tumor cells subcutaneously on day 0 and then untreated (Ctrl) or vaccinated with CPQ/Nes2LR, 2HPQ/Nes2LR on day 10 and 17. Mice were sacrificed on day 22, spleens and tumors were collected for analysis. **A**) Percentage of tet⁺CD8⁺ T cells in live cells. **B**) Number of tet⁺CD8⁺ T cell in 100,000 cells. Error bars show mean +/−std. dev. for n=5 mice per group. ** $p < 0.01$, and **** $p < 0.0001$, analyzed by two-way ANOVA with Tukey multiple comparisons post-test.

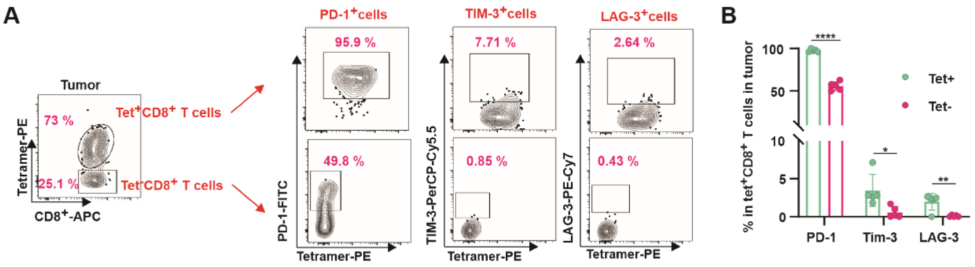

Figure S9. With the CPQ/Nes2LR vaccination, Nes2LR tet$^+$ T cells expressed higher percentage of PD-1, TIM-3 and LAG-3 compared to tet$^-$ T cells in tumor. BALB/c mice were inoculated with RENCA tumor cells subcutaneously on day 0 and then vaccinated with CPQ/Nes2LR on day 10 and 17. Mice were sacrificed on day 22, tumors were collected for analysis. **A**) Flow cytometry gating and percentage (**B**) of PD-1$^+$, TIM-3$^+$ and LAG-3$^+$ cells in the tet$^+$CD8$^+$ and tet$^-$CD8$^+$ T cell population. Error bars show mean +/−std. dev. for n=5 mice per group. * p < 0.05, ** p < 0.01, and **** p < 0.0001, analyzed by unpaired student t test.

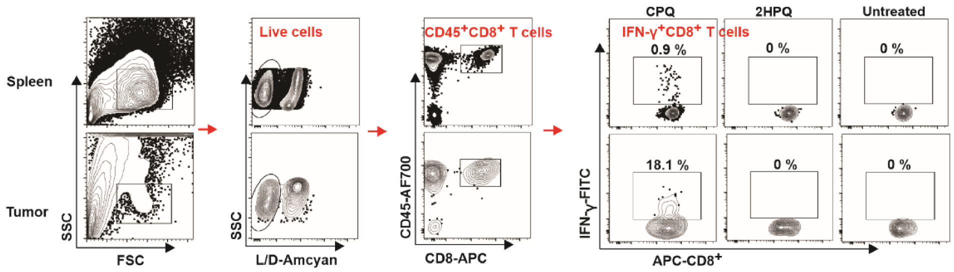

Figure S10. Flowcytometry gating of IFN-r producing cells in the CD8$^+$ T cell cells in spleen and tumor.

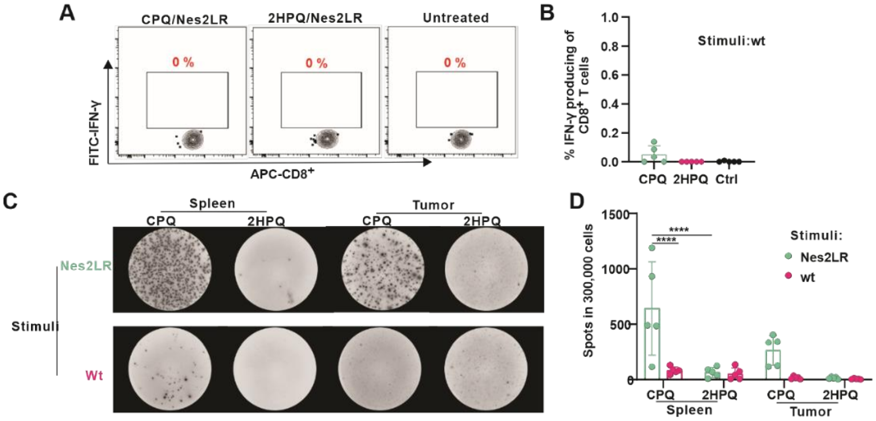

Figure S11. T cells induced by CPQ/Nes2LR (AYTTQREEL) vaccination are not reactive with the non-mutated epitope (AYTTQLEEL). BALB/c mice were inoculated with RENCA tumor cells subcutaneously on day 0 and then untreated or vaccinated with CPQ/Nes2LR, 2HPQ/Nes2LR on day 10 and 17. Mice were sacrificed on day 22, spleens and tumors were

collected for analysis. Splenocytes and TIL were stimulated with either 10 μg/mL Nes2LR or wildtype peptide *in vitro*. **A**) Flow cytometry gating and (**B**) percentage of IFN-γ producing cells in the $CD8^+$ T cell population after splenocyte stimulation with the non-mutated Nesprin epitope **C**) Images of ELISpot results. **D**) Summary of the ELISpot results. Error bars show mean +/−std. dev. for n=5 mice per group. **** p < 0.0001, analyzed by (D) two-way ANOVA with Tukey multiple comparisons post-test.

Table S1- RENCA peptides screened.

Name	Gene	Mut	Sequence	MHC allele	NetMHC percentile/ Rank
Renca_1	B4galnt1	G391A	3His-AYATTYRQL	H-2K^d	0.04
Renca_2	Nesprin-2	L4492R	3His-AYTTQREEL	H-2K^d	0.06
Renca_3	Mrps9	K384M	3His-EGARRMFTW	H-2D^d	0.01
Renca_4	Ipo9	A698V	3His-FPAVVQCTL	H-2L^d	0.1
Renca_5	Fnbp1	K315T	3His-GGTSRGKLW	H-2D^d	0.08
Renca_6	Tmem189	V202I	3His-GLPYWVTIL	H-2D^d	0.1
Renca_7	Pank2	L176I	3His-GYFGAVGAI	H-2K^d	0.06
Renca_8	Yme1l1	A539G	3His-GYHESGHAI	H-2K^d	0.01
Renca_9	Fndc3b	E728Q	3His-HGPQLECTV	H-2D^d	0.09
Renca_10	Actb	N280T	3His-IHETTFTSI	H-2K^d	0.07
Renca_11	Scap	F509V	3His-IYVLARTRL	H-2K^d	0.04
Renca_12	Mfsd1	A339T	3His-LYAVATTLV	H-2K^d	0.09
Renca_13	Rock2	A145P	3His-MPFANSPWV	H-2L^d	0.07
Renca_14	Pum3	E500Q	3His-SYLQGHTQQ	H-2K^d	0.06
Renca_15	Slc12a6	F254V	3His-SYVMISRAL	H-2K^d	0.04
Renca_16	mt-Co1	A303T	3His-TYFTSATMI	H-2K^d	0.01
Renca_17	Usp38	R327G	3His-VGPGALAVL	H-2D^d	0.025
Renca_18	Polr2j	A30T	3His-VPNTCLFTI	H-2L^d	0.025
Renca_19	Vat1	A165V	3His-VPSVQTFLM	H-2L^d	0.1
Renca_20	Edem3	E5425D	3His-YYLDVGKTL	H-2K^d	0.02

Table S2- RENCA tumor re-challenge results.

Treatment	Tumor formed post-challenge
Untreated	1/1
CPQ/Nes2LR	0/8
